# Association between serum biomarkers CEA and LDH and response in advanced non‐small cell lung cancer patients treated with platinum‐based chemotherapy

**DOI:** 10.1111/1759-7714.13449

**Published:** 2020-05-07

**Authors:** Corine de Jong, Vera H.M. Deneer, Johannes C. Kelder, Henk Ruven, Toine C.G. Egberts, Gerarda J.M. Herder

**Affiliations:** ^1^ Department of Clinical Pharmacy St. Antonius Hospital Nieuwegein/Utrecht The Netherlands; ^2^ Department of Clinical Pharmacy University Medical Center Utrecht Utrecht The Netherlands; ^3^ Division of Pharmacoepidemiology and Clinical Pharmacology, Department of Pharmaceutical Sciences, Faculty of Science Utrecht University Utrecht The Netherlands; ^4^ Department of Epidemiology and Statistics St. Antonius Hospital Nieuwegein/Utrecht The Netherlands; ^5^ Department of Clinical Chemistry St. Antonius Hospital Nieuwegein/Utrecht The Netherlands; ^6^ Department of Pulmonology Meander Medical Center Amersfoort The Netherlands

**Keywords:** Non‐small cell lung cancer (NSCLC), small cell lung cancer (SCLC), carcinoembryonic antigen (CEA), lactate dehydrogenase (LDH), noninvasive quantification of tumor biomarkers

## Abstract

**Background:**

In addition to radiological evaluation, biomarkers may be useful in providing early information on the response to treatment, and supporting clinical decision‐making. The objective of this study was to investigate carcinoembryonic antigen (CEA) and lactate dehydrogenase (LDH) as biomarkers for early assessment of response in patients with advanced non‐small cell lung cancer (NSCLC) treated with platinum‐based chemotherapy.

**Methods:**

A retrospective follow‐up study was conducted from 2012 to 2017 among 593 consecutive patients with advanced NSCLC treated with first‐line platinum‐based chemotherapy in a large teaching hospital in the Netherlands. Pretreatment biomarker levels and changes from pretreatment levels were studied for association with radiologic response (partial response [PR] or complete response [CR], according to RECIST 1.1) using multivariate logistic regression, and with overall survival using COX proportional hazard modeling. Patient and disease characteristics such as age and disease stage were taken into account as potential confounding factors.

**Results:**

Decreases in CEA and LDH (≥ 20%), particularly early in treatment, were significantly associated with better radiological response. Increases in these biomarkers (≥ 20%) and high pretreatment LDH levels (≥ 247 U/L) were significantly associated with lower overall survival.

**Conclusions:**

Our results support determination of CEA and LDH levels for earlier assessment of response to platinum‐based chemotherapy in patients with advanced NSCLC. Hence, routine determination and evaluation of CEA and LDH levels, prior to each cycle of platinum‐based chemotherapy in advanced NSCLC, should be considered as part of daily clinical practice.

**Key points:**

**Significant findings of the study:**

- Serum biomarkers in monitoring of treatment in advanced NSCLC would be useful.
- CEA and LDH decrease (≥ 20%) is favorable for achieving radiological response.
- High LDH levels and CEA/LDH increase (≥ 20%) is associated with reduced survival.

**What this study adds:**

- Monitoring of CEA seems to be particularly relevant in early stage of treatment.
- CEA and LDH determination should be considered as part of daily clinical practice.

## Introduction

Platinum‐based chemotherapy, often combined with immunotherapy in current practice, is the most frequently applied first‐line treatment for patients with advanced non‐small cell lung cancer (NSCLC) without an epidermal growth factor receptor (*EGFR*) mutation or anaplastic lymphoma kinase (ALK) rearrangement.^1^, ^2^ However, the added value of chemotherapy is limited compared with best supportive care, given the median survival benefit of less than three months and the substantial impact of chemotherapy‐induced toxicity on quality of life.^3^, ^4^, ^5^ Since clearly not all patients will benefit from systemic chemotherapy, early evaluation of response to treatment is of great relevance. The measurement of treatment response by radiological evaluation, takes place after two and four cycles of platinum‐based treatment.^6^ Thus, a first evaluation is feasible six and 12 weeks after treatment initiation. Serum biomarkers predicting response earlier in treatment would be useful in addition to standard clinical imaging methods.

Carcinoembryonic antigen (CEA), a glycoprotein involved in the modulation of cellular processes, cell‐cell recognition and cell adhesion, is used worldwide as a biomarker in several malignancies.^7^ Data from a few studies have suggested that pretreatment CEA levels and changes from pretreatment levels during treatment are indicative of treatment response in lung cancer.^8^, ^9^, ^10^ However, these results were obtained from small cohorts of patients which differ largely e.g. in terms of stages of disease. Another biomarker used in the follow‐up of cancer treatment is lactate dehydrogenase (LDH), an enzyme that plays an essential role in anaerobic glycolysis and induces cell proliferation. As higher LDH levels are associated with the promotion of tumor invasion and metastases, high LDH levels indicate poor overall survival in NSCLC.^11^, ^12^, ^13^

Current clinical guidelines regarding the monitoring of treatment in advanced lung cancer do not recommend the routine determination of biomarkers.^6^ To evaluate CEA and LDH levels in relation to treatment response, a retrospective follow‐up study in a large cohort of patients with advanced NSCLC receiving first‐line platinum‐based chemotherapy, was conducted.

## Methods

### Study population

This retrospective follow‐up study with prospectively collected data was conducted in a teaching hospital in the Netherlands (St. Antonius Hospital, Nieuwegein/Utrecht) in which approximately 200 patients are newly‐diagnosed with NSCLC yearly. Consecutive patients with pathology proven advanced NSCLC (stage IIIA, IIIB, or IV, according to tumor node metastasis [TNM] version 7) who started with first‐line platinum‐based (cisplatin or carboplatin) chemotherapy according to the ESMO Clinical Practice Guidelines between 01 January 2012 and 31 December 2017 were eligible.^6^, ^14^ Patients diagnosed with mesothelioma, patients who underwent lobectomy with adjuvant chemotherapy in stage IIIA, and patients with missing pretreatment levels of both CEA and LDH were excluded. Serum CEA and LDH levels were determined to a maximum of one month prior to start chemotherapy, and prior to each platinum‐based chemotherapy cycle, which is part of the hospital's standard of care for the entire population of NSCLC patients. The study was conducted in accordance with the guidelines for the REporting of tumor MARKer studies (REMARK).^15^ All data were extracted from the hospital's electronic medical record system.

### Ethical considerations

The study protocol complied with the Good Clinical Practice Guidelines and the Declaration of Helsinki (64th WMA General Assembly, Fortaleza, Brazil, October 2013). The hospital's accredited Medical Ethics Committee assessed the study protocol and concluded that the Human Subjects Act (Dutch legislation: WMO) did not apply to this study. Consequently, the committee officially stated to having no objection to the conduct of the study followed by the board of directors of our hospital giving written permission for the conduct of the study. All patients gave permission for the use for research purposes of (coded) data collected as part of regular patient care. The inclusion in the study did not change patients' care they received or additional interventions such as blood sampling.

### Assessment of treatment response

Treatment response was assessed radiologically and in terms of survival. Radiological response to treatment was measured after two and four chemotherapy cycles (at six and 12 weeks after treatment initiation, respectively) by computed tomography (CT) scan, fluorine‐18 deoxyglucose positron emission tomography (FDG‐PET) and/or magnetic resonance imaging (MRI) and assessed by pulmonary physicians specialized in pulmonary oncology. Response was categorized as progressive disease (PD), stable disease (SD), partial response (PR) or complete response (CR), according to the World Health Organization (WHO) Response Evaluation Criteria in Solid Tumors (RECIST 1.1).^16^ Pretreatment tumor assessment was performed by chest CT imaging. For this study, overall response rate was used and patients were classified as either “responder” (PR or CR) or “non‐responder” (PD or SD) to therapy, at six and 12 weeks after platinum‐based chemotherapy initiation.

Individual patient overall survival time was defined as the time difference between the date of pretreatment biomarker measurement until death. The last extraction of data from the medical records was performed on 31 January 2019. Patients who were alive had their data censored at the last date of contact, as reported in the medical record.

### Analysis of CEA and LDH

Measurements of CEA and LDH were performed by the Department of Clinical Chemistry of the St. Antonius Hospital in Nieuwegein/Utrecht, The Netherlands, using standardized diagnostic methods on an automated Cobas 6000 platform (Roche Diagnostics, Mannheim, Germany). CEA levels were measured using an electrochemiluminescence immunoassay (Roche Diagnostics). LDH measurements were performed using the IFCC‐recommended enzymatic assay of Roche Diagnostics (LDHI2). Internal and external (interlaboratory comparisons) quality control procedures were in place. For internal quality control procedures, two levels of Liquichek Unassayed Chemistry Control (for LDH) and Liquicheck Immunoassay plus (for CEA) were used (Bio‐Rad, Hercules, CA, USA) daily. Analytical performance based on the external quality control system for LDH was as follows; bias of 3.5% and a, precision of 4.3%, yielding a total measurement uncertainty of 12.1%. For CEA, the bias was 0.2% and the precision 5.7%, with a total measurement uncertainty of 11.6%.

### Potential confounding variables

The following parameters were considered to be potentially confounding variables: gender, age at diagnosis, Eastern Cooperative Oncology Group (ECOG) performance status (on a 5‐point scale, with higher scores indicating increasing disability),^17^ histological tumor type (NSCLC squamous cell, NSCLC nonsquamous and SCLC), disease stage, number of cycles of first‐line platinum‐based chemotherapy, smoking status, pretreatment LDH level, and manifestation of metastases in the central nervous system (CNS). CNS metastases (at diagnosis or within 30 days after diagnosis) were determined by CT or MRI scan.

### Data analysis

Statistical analysis was performed using SPSS version 25.0 (IBM SPPS Statistics), R version 3.2.1 (www.r-project.org), and GraphPad Prism 8.0.1. Standard summary statistics were used to describe the sample data set.

High pretreatment biomarker level was defined as any value above the local upper limit of normal, i.e., CEA levels ≥ 5.0 μg/L for non‐smokers, ≥ 10.0 μg/L for smokers and LDH levels ≥ 247 U/L. Changes in biomarker levels from pretreatment levels were calculated at three, six, nine and 12 weeks. To differentiate patients with and without biomarker change, and to indicate whether levels decreased or increased, the population was divided into three categories: “decreased” (biomarker level decrease ≥ 20%), “unchanged” (biomarker level decrease < 20% or biomarker level increase < 20%) and “increased” (biomarker level increase ≥ 20%), based on earlier published cutoff values for biomarker response.^9^

The strength of the association between biomarker levels (i.e., pretreatment levels and changes from pretreatment levels during treatment) and radiological response was estimated using logistic regression and expressed as odds ratios (OR) with 95% confidence intervals (CI). Median overall survival was plotted in Kaplan‐Meier curves and groups were compared by using the log rank test. Hazard ratios (HR) with 95% CI were calculated with Cox proportional hazard modeling. The multivariate setting of both logistic regression and Cox proportional hazard regression was used to take all potential confounders into account and to calculate adjusted OR (ORadj) and adjusted HR (HRadj). Age, ECOG PS and LDH pretreatment level were categorized into two groups (≤ 65 and > 65 years, ECOG PS 0–1 and ≥ 2, and LDH < 247 U/L and ≥ 247 U/L, respectively), and included in multivariate analysis.

## Results

### Patient characteristics

A total of 593 consecutive patients with previously untreated advanced NSCLC, receiving platinum‐based chemotherapy, between 01 January 2012 and 31 December 2017 were retrospectively screened for inclusion. In total 486 patients were included (107 patients were excluded: 104 patients underwent lobectomy; two patients were diagnosed with mesothelioma and one patient had missing pretreatment CEA and LDH levels).

The majority of the study population was male (55.1%), and the median age at diagnosis was 64 years (range: 33–84 years) (Table 1). The population included 138 patients (28.4%) diagnosed with SCLC and 348 (71.6%) with NSCLC, of which 235 (67.5%) had the nonsquamous histologic subtype. At diagnosis, 67 patients (13.7%) had manifestation of metastases in the CNS. In total, 432 (88.8%) were active smokers or had smoked in the past. Before treatment initiation, the vast majority of patients (90.4%) had an ECOG PS score of 0 or 1. All patients received at least one cycle of first‐line platinum‐based chemotherapy, 376 (77.4%) patients received three or four cycles until 12 weeks after treatment initiation. High pretreatment CEA and LDH levels were found in 254 (52.3%) and 232 (47.7%) patients, respectively.

**Table 1:** Patient characteristics

Characteristics	*n* (%)
Number of patients	486 (100)
Gender (male)	268 (55.1)
Age at diagnosis (years)	
Median (range)	64 (33–84)
> 65 years	188 (38.7)
Tumor histology	
NSCLC	348 (71.6)
Nonsquamous	235 (67.5)
Squamous	82 (23.6)
Large cell	23 (6.6)
Combined or unspecified	8 (2.3)
SCLC	138 (28.4)
Disease stage	
IIIA	94 (19.3)
IIIB	87 (17.9)
IV	305 (62.8)
CNS metastases (at diagnosis)	67 (13.7)
Cycles of platinum‐based chemotherapy	
1	40 (8.2)
2	70 (14.4)
3	151 (31.1)
4	225 (46.3)
Performance status	
ECOG 0	126 (26.0)
ECOG 1	313 (64.4)
ECOG ≥2	40 (8.2)
Unknown	7 (1.4)
Smoking status	
Never	44 (9.1)
Active	177 (36.4)
Former	255 (52.4)
Unknown	10 (2.1)
CEA pretreatment levels (μg/L)	
Available levels	454 (93.4)
Median (IQR)	6.5 (2.7–28)
High (≥ 5.0 μg/L (non‐smokers), ≥ 10.0 μg/L (smokers))	254 (52.3)
LDH pretreatment levels (U/L)	
Available levels	486 (100)
Median (IQR)	244 (202–317)
High (≥ 247 U/L)	232 (47.7)

CEA, carcinoembryonic antigen; CNS, central nervous system; ECOG PS, Eastern Cooperative Oncology Group performance status; IQR, interquartile range; LDH, lactate dehydrogenase; NSCLC, non‐small cell lung cancer; SCLC, small cell lung cancer.

### Radiological response

At six and 12 weeks after platinum‐based chemotherapy initiation, 240 (49.4%) respectively 188 (38.7%) patients showed radiological response (PR or CR). Radiological evaluation revealed statistically significant (*P* < 0.001) differences in response between tumor histology at week 6 (NSCLC 41.1% vs. SCLC 70.3%) and week 12 (NSCLC 30.7% vs. SCLC 58.7%). Stratified analysis of histology subtypes for the association between pretreatment biomarker levels and changes from pretreatment levels and radiological response did not show differences (data not shown). In addition, the number of cycles of platinum‐based chemotherapy was significantly associated with radiological response at week 6, but not at week 12 (Appendix S1, Table A).

As shown in Tables 2 and 3, high pretreatment CEA levels and high LDH levels were not associated with radiological response. Multivariate analyses demonstrated, particularly in early stage of treatment, significant associations between CEA decreases and favorable response. Significant associations were found between CEA decrease at week 3 and radiological response (CR and PR) at week 6 (ORadj 2.27, 95% CI: 1.28–4.03), and between CEA decrease at week 6 and better response at week 6 (ORadj 2.38, 95% CI: 1.36–4.17). Also CEA decrease at week 3 and favorable response at week 12 were associated (ORadj 2.09, 95% CI: 1.14–3.83). Significant associations were found between LDH decrease at week 3 and response at week 6 (ORadj 1.72, 95% CI: 1.02–2.88) and LDH decrease at week 6 and response at week 6 (ORadj 1.82, 95% CI: 1.07–3.09).

**Table 2:** Association between CEA levels and radiological response

		Week 6			Week 12	
Radiological response (PR or CR)		Univariate analysis	Multivariate analysis†		Univariate analysis	Multivariate analysis†
Biomarker levels CEA	N	Crude odds ratio (95% CI)	Adjusted odds ratio (95% CI)	N	Crude odds ratio (95% CI)	Adjusted odds ratio (95% CI)
Low pretreatment < 5.0 μg/L (non‐smokers) < 10.0 μg/L (smokers)	182	1 (ref)	1 (ref)	165	1 (ref)	1 (ref)
High pretreatment ≥ 5.0 μg/L (non‐smokers) ≥ 10.0 μg/L (smokers)	233	0.72 (0.48–1.06)	0.68 (0.43–1.07)	211	0.90 (0.60–1.36)	0.92 (0.57–1.49)
Week 0 and 3						
Unchanged < 20% decreased / < 20% increased	210	1 (ref)	1 (ref)	189	1 (ref)	1 (ref)
Increased ≥ 20%	90	1.50 (0.91–2.46)	1.54 (0.90–2.65)	80	1.16 (0.69–1.97)	1.21 (0.66–2.23)
Decreased ≥ 20%	86	2.50 (1.47–4.24)	2.27 (1.28–4.03)	83	2.51 (1.48–4.29)	2.09 (1.14–3.83)
Week 0 and 6						
Unchanged < 20% decreased / < 20% increased	133	1 (ref)	1 (ref)	126	1 (ref)	1 (ref)
Increased ≥ 20%	113	1.05 (0.63–1.73)	1.11 (0.64–1.93)	102	0.74 (0.44–1.27)	0.78 (0.43–1.43)
Decreased ≥ 20%	121	2.23 (1.35–3.71)	2.38 (1.36–4.17)	112	1.93 (1.15–3.24)	1.79 (1.00–3.20)
Week 0 and 9						
Unchanged < 20% decreased / < 20% increased	‐	‐	‐	85	1 (ref)	1 (ref)
Increased ≥ 20%	‐	‐	‐	75	0.72 (0.39–1.35)	0.80 (0.40–1.62)
Decreased ≥ 20%	‐	‐	‐	145	1.23 (0.72–2.10)	1.18 (0.64–2.16)
Week 0 and 12						
Unchanged < 20% decreased / < 20% increased	‐	‐	‐	69	1 (ref)	1 (ref)
Increased ≥ 20%	‐	‐	‐	63	0.81 (0.41–1.62)	0.81 (0.36–1.82)
Decreased ≥ 20%	‐	‐	‐	113	1.51 (0.83–2.76)	1.36 (0.68–2.71)

† Adjusted odds ratio: adjusted for gender, age, ECOG PS, histological subtype (NSCLC squamous, NSCLC nonsquamous, SCLC), cancer stage, number of cycles of first‐line platinum‐based chemotherapy, CNS metastasis, smoking history and pretreatment LDH level in multivariate logistic regression.

**Table 3:** Association between LDH levels and radiological response

		Week 6			Week 12	
Radiological response (PR or CR)		Univariate analysis	Multivariate analysis†		Univariate analysis	Multivariate analysis†
Biomarker levels LDH	N	Crude odds ratio (95% CI)	Adjusted odds ratio (95% CI)	N	Crude odds ratio (95% CI)	Adjusted odds ratio (95% CI)
Low pretreatment < 247 U/L	234	1 (ref)	1 (ref)	215	1 (ref)	1 (ref)
High pretreatment ≥ 247 U/L	211	1.12 (0.77–1.63)	1.04 (0.69–1.58)	189	1.04 (0.71–1.54)	0.93 (0.59–1.45)
Week 0 and 3						
Unchanged < 20% decreased/< 20% increased	249	1 (ref)	1 (ref)	229	1 (ref)	1 (ref)
Increased ≥ 20%	58	1.15 (0.65–2.04)	1.40 (0.75–2.62)	52	0.86 (0.47–1.58)	1.12 (0.57–2.24)
Decreased ≥ 20%	130	2.10 (1.35–3.26)	1.72 (1.02–2.88)	115	1.48 (0.95–2.33)	1.07 (0.61–1.85)
Week 0 and 6						
Unchanged < 20% decreased/< 20% increased	210	1 (ref)	1 (ref)	189	1 (ref)	1 (ref)
Increased ≥ 20%	72	1.14 (0.67–1.95)	1.25 (0.69–2.25)	63	1.04 (0.59–1.86)	1.00 (0.53–1.90)
Decreased ≥ 20%	143	2.26 (1.46–3.51)	1.82 (1.07–3.09)	135	1.74 (1.11–2.72)	1.24 (0.70–2.17)
Week 0 and 9						
Unchanged < 20% decreased/< 20% increased	‐	‐	‐	152	1 (ref)	1 (ref)
Increased ≥ 20%	‐	‐	‐	61	1.48 (0.82–2.70)	2.06 (1.05–4.05)
Decreased ≥ 20%	‐	‐	‐	140	2.23 (1.40–3.57)	1.68 (0.92–3.06)
Week 0 and 12						
Unchanged < 20% decreased/<20% increased	‐	‐	‐	140	1 (ref)	1 (ref)
Increased ≥ 20%	‐	‐	‐	48	0.57 (0.29–1.15)	0.66 (0.31–1.43)
Decreased ≥ 20%	‐	‐	‐	103	1.83 (1.09–3.06)	1.43 (0.70–2.92)

† Adjusted odds ratio: adjusted for gender, age, ECOG PS, histological subtype (NSCLC squamous, NSCLC nonsquamous, SCLC), cancer stage, number of cycles of first‐line platinum‐based chemotherapy, CNS metastasis, smoking history and pretreatment LDH level in multivariate logistic regression.

### Survival analysis

**Figure 1:**
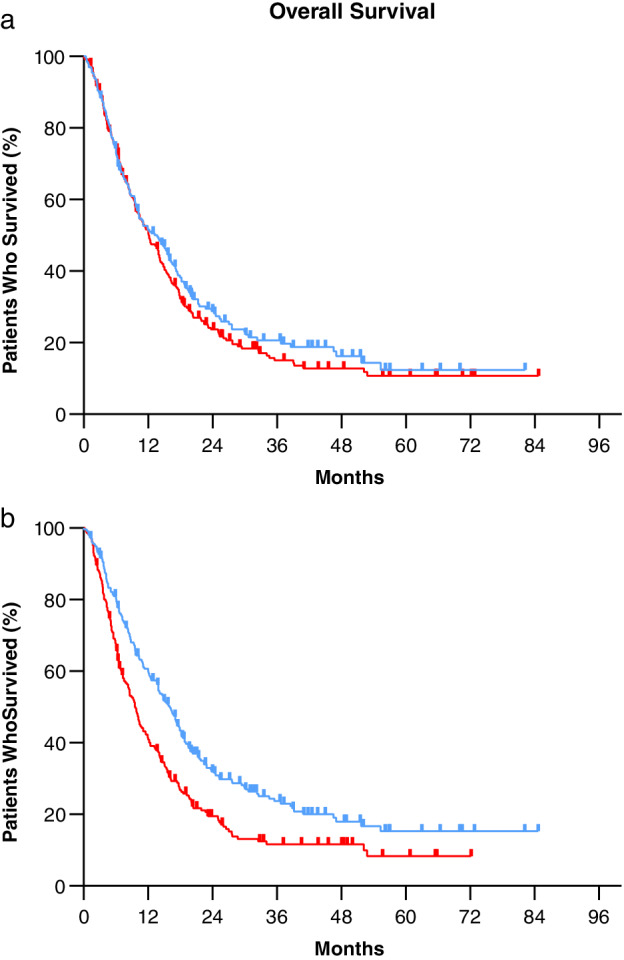
Overall survival. Kaplan‐Meier plots illustrate overall survival according to pretreatment CEA and LDH serum levels. (**a**) Pretreatment CEA levels. High pretreatment CEA levels defined as ≥ 5.0 μg/L (non‐smokers) and ≥ 10 μg/L (smokers) (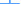) Low pretreatment CEA levels, (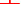) High pretreatment CEA levels. (**b**) Pretreatment LDH levels. High pretreatment LDH levels defined as ≥ 247 U/L (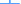) Low pretreatment LDH levels, (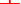) High pretreatment LDH levels. Blue lines indicate patients with low pretreatment biomarker levels and red lines indicate those with high levels. Overall survival is calculated in months after pretreatment biomarker measurement until death. Hazard ratios were calculated in univariate setting with Cox proportional hazard modeling. Abbreviations: CEA, carcinoembryonic antigen; CI, confidence interval; HR, hazard ratio; LDH, lactate dehydrogenase.

Median follow‐up duration from pretreatment biomarker measurement was 11.4 months (interquartile range [IQR] 5.5–20.3 months) with a median overall survival for the total cohort of 12.2 months (95% CI: 10.4–14.0). ECOG PS, disease stage, number of cycles of first‐line platinum‐based chemotherapy, and pretreatment LDH level were significantly associated with overall survival **(**Fig. 1, Appendix S1, Table B **)**.

No statistically significant differences in overall survival between patients with NSCLC and SCLC were found (12.5 vs. 10.6 months respectively). In addition, stratified analysis of histology subtypes for the association between pretreatment biomarker levels and changes from pretreatment levels and overall survival did not show differences (data not shown). As shown in Table 4, multivariate analyses demonstrated that CEA increases at week 3 (HRadj 1.70, 95% CI: 1.27–2.27) and week 6 (HRadj 1.44, 95% CI: 1.07–1.95), were negatively associated with overall survival. High pretreatment LDH level (HRadj 1.42, 95% CI: 1.15–1.76), LDH increases at week 3 (HRadj 1.62, 95% CI: 1.18–2.22), week 6 (HRadj 1.47, 95% CI: 1.08–2.00) and week 12 (HRadj 1.71, 95% CI: 1.15–2.54) were associated with reduced overall survival (Fig. 1, Table 5).

**Table 4:** Association between CEA levels and overall survival

Overall survival			Univariate analysis	Multivariate analysis†
Variable	N	Median (months) (95% CI)	Hazard ratio (95% CI)	Hazard ratio (95% CI)
Total cohort	486	12.2 (10.4–14.0)	‐	‐
Biomarker levels CEA				
Low pretreatment < 5.0 μg/L (non‐smokers) < 10.0 μg/L (smokers)	200	13.2 (9.8–16.6)	1 (ref)	1 (ref)
High pretreatment ≥ 5.0 μg/L (non‐smokers) ≥ 10.0 μg/L (smokers)	254	12.1 (10.1–14.1)	1.10 (0.89–1.36)	1.07 (0.85–1.35)
Week 0 and 3				
Unchanged < 20% decreased / < 20% increased	219	14.8 (12.8–16.8)	1 (ref)	1 (ref)
Increased ≥ 20%	96	8.1 (5.7–10.5)	1.65 (1.26–2.16)	1.70 (1.27–2.27)
Decreased ≥ 20%	91	14.5 (11.5–17.5)	1.00 (0.76–1.32)	0.91 (0.68–1.22)
Week 0 and 6				
Unchanged < 20% decreased/< 20% increased	137	15.6 (13.0–18.2)	1 (ref)	1 (ref)
Increased ≥ 20%	115	8.6 (6.6–10.6)	1.51 (1.13–2.00)	1.44 (1.07–1.95)
Decreased ≥ 20%	124	16.4 (13.6–19.2)	0.96 (0.73–1.27)	0.86 (0.64–1.16)
Week 0 and 9				
Unchanged < 20% decreased/< 20% increased	93	15.6 (12.3–18.9)	1 (ref)	1 (ref)
Increased ≥ 20%	80	9.5 (7.2–11.8)	1.51 (1.07–2.13)	1.38 (0.95–2.00)
Decreased ≥ 20%	154	17.1 (15.4–18.8)	0.95 (0.71–1.29)	0.89 (0.64–1.24)
Week 0 and 12				
Unchanged < 20% decreased / < 20% increased	73	15.3 (13.6–17.0)	1 (ref)	1 (ref)
Increased ≥ 20%	65	10.8 (7.1–14.5)	1.07 (0.73–1.59)	0.91 (0.59–1.42)
Decreased ≥ 20%	118	15.4 (13.0–17.8)	1.00 (0.72–1.40)	0.93 (0.65–1.33)

Medians were calculated using the Kaplan‐Meier method. Hazard ratios were calculated in univariate and multivariate setting with Cox proportional hazard modeling.

† Multivariate analysis adjusted for gender, age, ECOG PS, histological subtype (NSCLC squamous, NSCLC nonsquamous, SCLC), cancer stage, number of cycles of first‐line platinum‐based chemotherapy, CNS metastasis, smoking history and pretreatment LDH level.

**Table 5:** Association between LDH levels and overall survival

Overall survival			Univariate analysis	Multivariate analysis†
Variable	N	Median (months) (95% CI)	Hazard ratio (95% CI)	Hazard ratio (95% CI)
Total cohort	486	12.2 (10.4–14.0)	‐	‐
Biomarker levels LDH				
Low pretreatment < 247 U/L	254	16.0 (14.0–18.0)	1 (ref)	1 (ref)
High pretreatment ≥ 247 U/L	232	9.5 (8.2–10.8)	1.53 (1.25–1.87)	1.42 (1.15–1.76)
Week 0 and 3				
Unchanged < 20% decreased / < 20% increased	268	15.6 (13.4–17.8)	1 (ref)	1 (ref)
Increased ≥ 20%	80	6.7 (3.6–9.8)	1.87 (1.39–2.52)	1.62 (1.18–2.22)
Decreased ≥ 20%	136	10.1 (8.2–12.0)	1.39 (1.10–1.76)	1.01 (0.78–1.32)
Week 0 and 6				
Unchanged < 20% decreased / < 20% increased	215	15.3 (12.9–17.7)	1 (ref)	1 (ref)
Increased ≥ 20%	78	9.7 (6.3–13.1)	1.42 (1.06–1.90)	1.47 (1.08–2.00)
Decreased ≥ 20%	148	11.9 (8.8–15.0)	1.20 (0.95–1.52)	0.83 (0.62–1.09)
Week 0 and 9				
Unchanged < 20% decreased / < 20% increased	161	16.7 (14.5–18.9)	1 (ref)	1 (ref)
Increased ≥ 20%	73	13.9 (9.7–18.1)	1.16 (0.83–1.60)	1.11 (0.78–1.59)
Decreased ≥ 20%	151	12.6 (9.5–15.7)	1.26 (0.98–1.63)	1.05 (0.78–1.42)
Week 0 and 12				
Unchanged < 20% decreased / < 20% increased	148	16.7 (14.1–19.3)	1 (ref)	1 (ref)
Increased ≥ 20%	51	13.8 (8.7–18.9)	1.46 (1.01–2.10)	1.71 (1.15–2.54)
Decreased ≥ 20%	108	11.5 (8.5–14.5)	1.54 (1.15–2.05)	1.36 (0.96–1.94)

Medians were calculated using the Kaplan‐Meier method. Hazard ratios were calculated in univariate and multivariate setting with Cox proportional hazard modeling.

† Multivariate analysis adjusted for gender, age, ECOG PS, histological subtype (NSCLC squamous, NSCLC nonsquamous, SCLC), cancer stage, number of cycles of first‐line platinum‐based chemotherapy, CNS metastasis, smoking history and pretreatment LDH level.

## Discussion

This study reveals that decreases (≥ 20%) in CEA and LDH levels, especially those early in treatment, are associated with favorable radiological response to platinum‐based chemotherapy in previously untreated advanced‐stage lung cancer. In addition, increases in these biomarkers (≥ 20%) and pretreatment high LDH are associated with lower overall survival. In the current study, biomarker response was divided into three categories, which made it possible to distinguish patients with decreased (≥ 20%) biomarker levels as well as patients with unchanged (< 20% decrease/increase) and increased (≥ 20%) biomarker levels. As compared with a decrease in LDH level, a decrease in CEA level at week 3 was found to be stronger associated with better radiological response at week 6 (1.7‐ and 2.3‐fold higher probability, respectively). Since the association between CEA level decrease with radiological response is already shown after the first cycle of chemotherapy, monitoring of CEA levels seems to be particularly relevant in early stage of treatment. Pretreatment levels of CEA and LDH were not associated with radiological response. However, CEA and LDH increase at week 3, as compared with unchanged or decreased biomarker levels, was associated with a significant 1.7‐ and 1.6‐fold higher probability of reduced overall survival. In addition, a 1.4‐fold higher probability of inferior overall survival was found in patients with high pretreatment LDH levels. These results are in line with previously reported data suggesting that LDH serum levels may be useful on predicting clinical outcome in patients treated with first‐line chemotherapy for different malignancies.^11^, ^12^, ^13^, ^18^, ^19^ For both biomarkers, changes during treatment were superior to pretreatment biomarker levels in predicting therapy response, advocating biomarker assessment during treatment follow‐up.

These findings support the results of an earlier published systemic review and meta‐analysis.^20^ According to Holdenrieder and colleagues, changes from pretreatment CEA levels during treatment are indicative of treatment response in NSCLC. However, in our cohort biomarker level measurements were available after the first cycle of platinum‐based chemotherapy, while most studies report biomarker levels after the second cycle of chemotherapy. Therefore, detailed information earlier in treatment was provided in our cohort. Besides, due to the use of small study cohorts, the inclusion of patients with different stages of NSCLC and the use of different response classifications, the meta‐analysis of Holdenrieder *et al*. was influenced by a high level of between‐study heterogeneity.^20^

### Clinical implications

Biomarkers of treatment response are particularly relevant early after treatment initiation, even prior to radiological evaluation. Moreover, determination of biomarkers might be even more useful in the evaluation of patients with a mixed radiological response. Clinicians are also frequently confronted with patients with radiologically confirmed progressive disease accompanied with a beneficial clinical response and performance score or vice versa. In these cases, clinicians and patients are facing the dilemma of treatment (dis)continuation. Therefore, in addition to radiological evaluation, changes in biomarker levels might support the process of evaluating treatment response in the continuous consideration of harm and benefit. Currently, LDH measurement during treatment follow‐up is standard clinical care for advanced NSCLC.^6^ However, recommendations are lacking on how pretreatment LDH levels and changes should be taken into account in the assessment of response to platinum‐based chemotherapy. In addition, the results of our study indicate that CEA level changes are strongly associated with therapy response, supporting the recommendation that CEA and LDH assessment should be considered as part of standard of care for patients with previously untreated advanced NSCLC treated with platinum‐based chemotherapy.

### Strengths and limitations

The present study has several strengths. First, the biomarkers examined are routinely determined during treatment follow‐up of advanced NSCLC patients in our hospital. Therefore, the results of this study reflect the actual clinical setting.

Second, the study has a single‐center design. Since all patients were recruited in the same teaching hospital, low heterogeneity in clinical practice occurred, and all patients underwent the same treatment regimens. Besides, during the defined time frame, a large cohort of consecutive patients was formed, therefore avoiding selection bias. To our knowledge, this is the largest study conducted to investigate the association between CEA and LDH levels and treatment response in stage III/IV NSCLC. Additionally, the results can be implemented immediately into daily clinical practice, since measuring CEA and LDH levels is affordable and easy to perform.

The present analysis also has some limitations. First, the time of radiological evaluation was not predefined due to the retrospective nature of the study. CT scans were taken after two and four chemotherapy cycles, performed every six to eight weeks in routine care. Therefore, the first and second CT scan after treatment initiation was defined as radiological response at week 6 and 12, respectively. However, there was minor variation in the time of radiological evaluation. In addition, radiological response was measured by pulmonary physicians specialized in pulmonary oncology according to the RECIST 1.1 criteria. Since misclassification can occur, preferably, two observers should have evaluated the endpoints independently. On the other hand, our results reflect the actual clinical setting, a strength mentioned earlier.

### Future research

Based on our results, routine measurement and evaluation of both CEA and LDH levels should be considered as part of treatment evaluation in advanced lung cancer patients. However, to our knowledge, only a few hospitals in the Netherlands evaluate CEA levels during follow‐up of advanced NSCLC patients. Therefore, impact analysis of the implementation of routine biomarker determination on clinical decision‐making should be of special interest.

Despite the fact that platinum‐based chemotherapy has long been the standard first‐line treatment for patients with advanced NSCLC, the introduction of immunotherapy recently led to new treatment perspectives and strategies. Today, for patients with programmed cell death ligand 1 (PD‐L1) expression ≥50% of tumor cells (approximately one‐third of patients), immunotherapy or immunotherapy in combination with chemotherapy is the first‐line treatment option.^6^ For these patients starting with mono immunotherapy, recent studies already suggest the significance of both CEA and LDH for the assessment of treatment response,^21^, ^22^, ^23^, ^24^ which is in line with the findings presented here. Moreover, current research reveals the additional value of combining immunotherapy with platinum‐based chemotherapy as first‐line treatment.^25^, ^26^ Since patients in our cohort started with first‐line treatment between 01 January 2012 and 31 December 2017, the vast majority of our patients was treated with platinum‐based chemotherapy. Merely three patients (less than 1%) underwent chemotherapy combined with immunotherapy; hence subgroup analysis was not applicable. As determination of CEA and LDH levels in patients undergoing platinum‐based chemotherapy or immunotherapy proved to be relevant in treatment evaluation, it is likely that biomarker determination would also be appropriate in the follow‐up of combination therapy. Whether biomarker (changes) can also predict response in NSCLC patients undergoing novel targeted or immunotherapies combined with conventional chemotherapy, is an important topic for future research.

In conclusion, the results of this retrospective follow‐up study support the determination of both CEA and LDH serum levels for identifying subgroups of platinum‐based chemotherapy treated NSCLC patients differing in radiological response and overall survival. Hence, routine determination and evaluation of CEA and LDH levels, prior to each cycle of platinum‐based chemotherapy in advanced NSCLC, should be considered as part of daily clinical practice. Biomarker assessment might be particularly relevant alongside radiological evaluation, in the evaluation of patients with a mixed radiological response or in case of discrepancy between clinical and radiological responses.

## Disclosure

The authors have no conflicts of interest to declare.

## Supporting information


**Appendix** **S1**: Supplementary Material
