# Supplementary material for: Association between serum biomarkers CEA and LDH and response in advanced non‐small cell lung cancer patients treated with platinum‐based chemotherapy
Source: Thorac Cancer. 2020 May 7;11(7):1790–800. doi: 10.1111/1759-7714.13449 (PMC7327701; doi:10.1111/1759-7714.13449)
Supplement: Supplementary file 1 — Appendix S1: Supplementary Material [file TCA-11-1790-s001.docx]

**SUPPLEMENTARY APPENDIX**

**Table A: Univariate and multivariate analyses of radiological response, potential confounders**

| **Radiological response**  **(PR or CR)** |  | **Week 6** |  |  | **Week 12** |  |
| --- | --- | --- | --- | --- | --- | --- |
|  |  | **Univariate**  **analysis** | **Multivariate analysis^1^** |  | **Univariate**  **analysis** | **Multivariate**  **analysis^1^** |
| **Biomarker levels CEA^1^** | **N** | **Crude**  **Odds ratio**  **(95% CI)** | **Adjusted**  **Odds ratio**  **(95% CI)** | **N** | **Crude**  **Odds ratio**  **(95% CI)** | **Adjusted**  **Odds ratio**  **(95% CI)** |
| Male | 247 | 1.0 (ref) | 1.0 (ref) | 219 | 1 (ref) | 1 (ref) |
| Female | 198 | 1.21 (0.83–1.76) | 1.25 (0.82–1.91) | 185 | 1.04 (0.70–1.54) | 0.98 (0.62–1.4) |
| Age ≤ 65 year | 272 | 1.0 (ref) | 1.0 (ref) | 251 | 1 (ref) | 1 (ref) |
| Age > 65 year | 173 | 0.95 (0.65–1.40) | 0.92 (0.59–1.42) | 153 | 1.17 (0.79–1.76) | 1.12 (0.70–1.78) |
| ECOG PS 0-1 | 404 | 1.0 (ref) | 1.0 (ref) | 368 | 1 (ref) | 1 (ref) |
| ECOG PS ≥ 2 | 36 | 0.96 (0.49–1.91) | 0.75 (0.35–1.63) | 31 | 0.22 (0.025–2.00) | 0.45 (0.19–1.08) |
| SCLC | 124 | 1.0 (ref) | 1.0 (ref) | 116 | 1 (ref) | 1 (ref) |
| NSCLC squamous | 75 | 0.27 (0.15–0.51) | 0.28 (0.15–0.55) | 64 | 0.28 (0.15–0.53) | 0.33 (0.16–0.68) |
| NSCLC non-squamous | 219 | 0.19 (0.12–0.32) | 0.20 (0.12–0.34) | 200 | 0.25 (0.16–0.41) | 0.24 (0.14–0.42) |
| NSCLC other | 27 | 0.41 (0.17–0.97) | 0.42 (0.17–1.03) | 24 | 0.22 (0.09–0.55) | 0.25 (0.09–0.67) |
| Stage IIIA | 83 | 1.0 (ref) | 1.0 (ref) | 76 | 1 (ref) | 1 (ref) |
| Stage IIIB | 83 | 1.05 (0.57–1.93) | 1.11 (0.58–2.14) | 75 | 1.66 (0.87–3.17) | 1.71 (0.84–3.47) |
| Stage IV | 279 | 1.05 (0.64–1.71) | 1.10 (0.63–1.92) | 253 | 1.36 (0.81–2.30) | 1.16 (0.61–2.20) |
| No CNS metastasis | 389 | 1.0 (ref) | 1.0 (ref) | 356 | 1 (ref) | 1 (ref) |
| CNS metastasis | 56 | 0.84 (0.48–1.46) | 0.66 (0.35–1.24) | 48 | 0.66 (0.35–1.22) | 0.56 (0.27–1.14) |
| Never smokers | 39 | 1.0 (ref) | 1.0 (ref) | 37 | 1 (ref) | 1 (ref) |
| Former smokers | 231 | 0.91 (0.46–1.81) | 0.71 (0.34–1.46) | 204 | 1.44 (0.71–2.93) | 1.30 (0.59–2.84) |
| Active smokers | 166 | 0.89 (0.44–1.80) | 0.78 (0.37–1.64) | 154 | 1.10 (0.53–2.28) | 1.14 (0.51–2.54) |
| 1 cycle platinum–based chemo | 31 | 1.0 (ref) | 1.0 (ref) | 7 | 1 (ref) | 1 (ref) |
| 2 cycle platinum–based chemo | 414 | 3.09 (1.39–6.86) | 2.70 (1.15–6.32) | 52 | 0.67 (0.11–3.94) | 0.82 (0.13–5.35) |
| 3 cycle platinum–based chemo | - | - | - | 125 | 1.90 (0.36–10.18) | 2.16 (0.37–12.58) |
| 4 cycle platinum–based chemo | - | - | - | 220 | 3.06 (0.58–16.09) | 3.88 (0.68–22.27) |
| Low pretreatment LDH levels (< 247 U/L) | 234 | 1.0 (ref) | 1.0 (ref) | 216 | 1 (ref) | 1 (ref) |
| High pretreatment LDH levels (≥ 247 U/L) | 211 | 1.12 (0.77–1.63) | 1.04 (0.69–1.58) | 88 | 1.04 (0.71–1.54) | 0.93 (0.59–1.45) |

**Table B:** **Univariate and multivariate analyses of overall survival, potential confounders**

| **Overall Survival** | | | **Univariate analysis** | **Multivariate analysis^1^** |
| --- | --- | --- | --- | --- |
| **Variable** | **N** | **Median (months)**  **(95% CI)** | **Hazard ratio**  **(95% CI)** | **Hazard ratio**  **(95% CI)** |
| Total cohort | 486 | 12.2 (10.4–14.0) | - | - |
| **Patient characteristics** |  |  |  |  |
| Male | 268 | 11.6 (9.1–14.1) | 1 (ref) | 1 (ref) |
| Female | 218 | 13.2 (10.7–15.7) | 0.86 (0.70–1.05) | 0.84 (0.68–1.04) |
| Age ≤ 65 year | 298 | 12.3 (10.2–14.4) | 1 (ref) | 1 (ref) |
| Age > 65 year | 188 | 12.2 (9.1–15.3) | 1.12 (0.91–1.37) | 1.11 (0.89–1.37) |
| ECOG PS 0-1 | 439 | 13.6 (11.9–15.3) | 1 (ref) | 1 (ref) |
| ECOG PS ≥ 2 | 40 | 7.6 (3.6–11.6) | 2.00 (1.43–2.87) | 1.68 (1.16–2.44) |
| SCLC | 138 | 10.6 (7.8–13.4) | 1 (ref) | 1 (ref) |
| NSCLC squamous | 82 | 12.2 (5.9–18.5) | 0.87 (0.63–1.18) | 0.96 (0.68–1.36) |
| NSCLC non-squamous | 235 | 13.8 (11.7–15.9) | 0.89 (0.70–1.13) | 0.92 (0.71–1.18) |
| NSCLC other | 31 | 10.0 (3.5–16.5) | 0.89 (0.57–1.39) | 0.82 (0.51–1.30) |
| Stage IIIA | 94 | 21.3 (16.8–25.8) | 1 (ref) | 1 (ref) |
| Stage IIIB | 87 | 17.7 (15.1–20.3) | 1.31 (0.92–1.86) | 1.69 (1.18–2.44) |
| Stage IV | 305 | 9.4 (8.0–10.8) | 2.16 (1.63–2.86) | 2.51 (1.83–3.45) |
| No CNS metastasis | 419 | 13.6 (11.9–15.3) | 1 (ref) | 1 (ref) |
| CNS metastasis | 67 | 6.7 (4.1–9.3) | 1.52 (1.14–2.02) | 1.23 (0.91–1.67) |
| Never smokers | 44 | 13.9 (11.1–16.7) | 1 (ref) | 1 (ref) |
| Former smokers | 255 | 11.6 (9.4–13.8) | 1.33 (0.92–1.93) | 1.16 (0.79–1.70) |
| Active smokers | 177 | 12.4 (8.8–16.0) | 1.27 (0.86–1.86) | 1.19 (0.80–1.77) |
| 1 cycle platinum–based chemo | 40 | 1.8 (1.5–2.1) | 1 (ref) | 1 (ref |
| 2 cycle platinum–based chemo | 70 | 5.5 (3.5–7.5) | 0.56 (0.37–0.84) | 0.42 (0.27–0.65) |
| 3 cycle platinum–based chemo | 151 | 17.7 (14.2–21.2) | 0.21 (0.14–0.30) | 0.19 (0.13–0.28) |
| 4 cycle platinum–based chemo | 225 | 15.0 (13.7–16.3) | 0.23 (0.16–0.33) | 0.14 (0.10–0.21) |
| Low pretreatment LDH levels (< 247 U/L) | 254 | 16.0 (14.0–18.0) | 1 (ref) | 1 (ref) |
| High pretreatment LDH levels (≥ 247 U/L) | 232 | 9.5 (8.2–10.8) | 1.53 (1.25–1.87) | 1.42 (1.15–1.76) |

^1^ Multivariate analysis adjusted for gender, age, ECOG PS, histological subtype (NSCLC squamous, NSCLC non-

squamous, SCLC), cancer stage, number of cycles of first-line platinum–based chemotherapy, CNS metastasis, smoking

history and pretreatment LDH level.
